# Synthesis, Characterization, and Polymerization of Ge- and Sn-Substituted [2.2]Paracyclophanes toward Poly(para-xylylene) Films and Their Mechanical Properties

**DOI:** 10.1021/acs.inorgchem.5c04945

**Published:** 2026-01-30

**Authors:** Moena Hirao, Lukas Bichlmaier, Tetsuhiko F. Teshima, Rebecca Wilhelm, Shigeyoshi Inoue

**Affiliations:** 1 TUM School of Natural Sciences, Department of Chemistry, Catalysis Research Center and Institute of Silicon Chemistry, Technical University of Munich, Lichtenbergstraße 4, 85748 Garching, Germany; 2 Medical & Health Informatics Laboratories NTT Research Incorporated, 940 Stewart Dr., Sunnyvale, California 94085, United States; 3 TUM School of Natural Sciences, Department of Chemistry and Catalysis Research Center, Chair of Technical Electrochemistry, Technical University of Munich, Lichtenbergstraße 4, 85748 Garching, Germany

## Abstract

Polymers have had widespread applications in industry over the past few decades. Recently, polymers incorporating heavier group 14 elements (Ge, Sn, and Pb) have gained interest since their oxides are promising for semiconductor applications due to their high dielectric constants and charge mobility. Poly­(*p*-xylylene) (PPX), an important class of polymer, is widely recognized for its transparency, biocompatibility, and the conformality afforded by its polymerization method, chemical vapor deposition (CVD). In this work, PPXs incorporating germanium or tin are prepared via CVD polymerization and the optimal pyrolysis temperatures of their precursors are determined. The ductility, thermal stability, crystallinity, surface topography, and comprehensive and surface chemical compositions are investigated. Sequential changes in the surface oxidation state are confirmed following exposure to air and subsequent oxygen plasma treatment. Comparison of the obtained PPXs with the widely applied chlorinated version PPX-Cl (trade name Parylene C) revealed preserved ductility while exhibiting distinct trends in softness.

## Graphical abstract

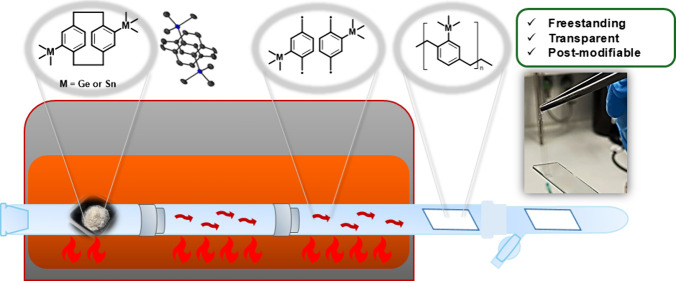

## Introduction

Polymers play fundamental roles in a wide range of industries, such as electronics and automotive. The most common hydrocarbon polymers, including polyethylene and polystyrene, have been widely used for decades for their durability, flexibility, and lightweight.^1^ Another important class of polymers, particularly polythiophenes, provides electrical conductivity,^2^ while polyamides exhibit high mechanical strength and chemical resistance.^3^

In the last few decades, chemical incorporation of heavier elements such as the carbon analogues, germanium (Ge) and tin (Sn), has captured much attention owing to their low electronegativities and, in some cases, inherent mechanical properties.^4,5^ While Ge and Sn can exhibit carbon-like electron configurations, molecular geometries, and bonding patterns, they also display distinct electronic properties and reactivities. Such similarities provide a strong rationale for investigating them as carbon analogues, particularly in reactions like nucleophilic substitution, oxidation, and cross-linking.^6^ Meanwhile, such heavier group 14 elements exhibit excellent properties as inorganic materials; their oxides are potentially valuable for semiconductor applications owing to the higher dielectric constants and electron-hole mobility, meaning they are promising gate dielectrics in transistors, enabling faster carrier transporting.^7–9^ In contrast to this, organic forms such as germoxanes (R_2_GeO)_*n*_ and stannoxanes (R_2_SnO)_*n*_ have garnered much interest due to their unique structural features and chemical properties.^10,11^ However, the number of such organic Ge–O and Sn–O systems remains relatively limited, especially within the scope of materials science.

Back to the heavy-element-incorporated organic polymers, their synthetic methods span a wide range of reactions such as single-electron reduction, ring-opening reactions, and metal-catalyzed coupling polymerizations, including Yamamoto, Wurtz, and Sonogashira couplings^12–16^ (Figure 1, compounds A–C). However, polymerizations are often performed in a solvent, which can result in residual solvent being trapped within the polymer matrix, thereby reducing the purity. Here, when focused on polymer films, a class of material with high industrial demand, the reported films deposited via dip or spin coating typically lack conformality and uniformity.^15,17,18^ A chemical vapor deposition (CVD) method overcomes such drawbacks, as the reactions are carried out in the gas phase and are applicable to substrates with any arbitrary geometry.^19^ Moreover, poly­(para-xylylene) (PPX) is a common polymer film deposited via CVD and is promising for its excellent transparency and dielectric property.^20^ Nevertheless, the fabrication of PPX with heavier elements has been scarcely investigated^21,22^ (Figure 1, compounds D–F). By integrating this historically significant polymer with the properties of methylgermane and methyltin, a freestanding, semiconductor-compatible organic polymer with a facile polymerization method was envisaged. Very recently, the incorporation of a carbon analogue into PPX, named PPX-SiMe_2_H (Figure 1, compound E), was reported by introducing dimethylsilane into the CVD precursor.^23^ In addition to its in situ siloxane formation during CVD, full surface oxidation to SiO_2_ was accomplished through oxygen plasma treatment. Here, the similar oxidation with the heavier group 14 homologues is considered plausible, the trend in bond dissociation energies (BDEs), which decrease in the order Si–H > Ge–H > Sn–H by ∼10 kcal/mol each, indicating that bond cleavage becomes increasingly facile with the heavier counterparts.^24^

**Figure 1:**
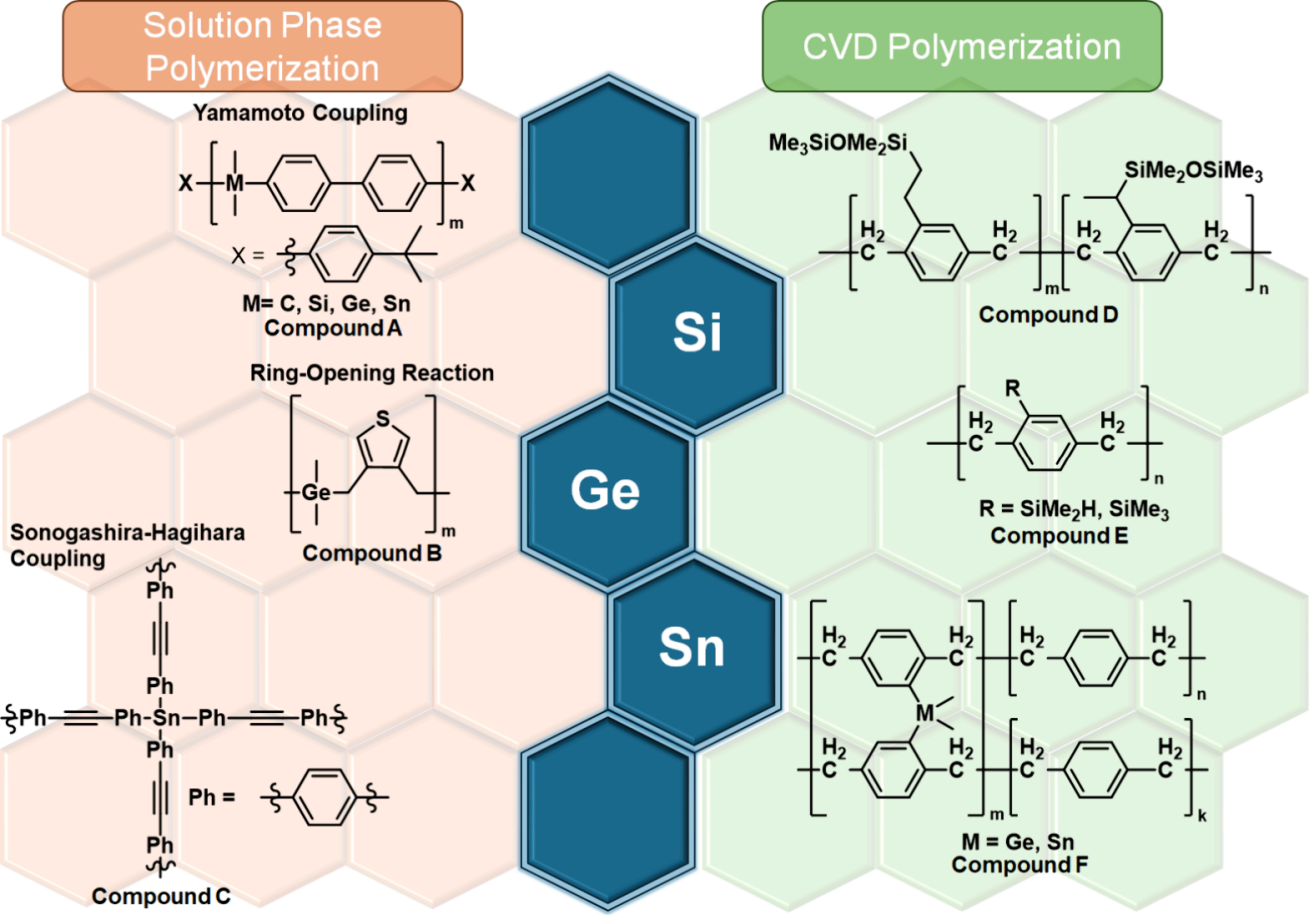
Polymers incorporating heavier group 14 elements. Left: polymers via solution-phase reaction. Right: polymers via the gas phase, CVD polymerization.

This work focuses on the synthesis of Ge- or Sn-incorporated PPX together with its oxidation and optimization of the polymerizing methodology. Through CVD polymerization and oxygen plasma treatment, novel PPXs incorporating a semiconductor-compatible oxide layer are obtained. Given that Ge–H and Sn–H compounds are generally air sensitive compared to Si–H, predominantly due to the mentioned BDEs, the CVD precursors with a Ge or Sn substituent with methyl groups (GeMe_3_ and SnMe_3_) are employed.^25^ The incorporation of Ge or Sn as substituents allows for facile synthesis and chemical stability together with the site-specific oxidation, unlike when these are directly incorporated into the backbone main chain.^26^ Although the polymerization and characterization of Ge- and Sn-containing PPXs were previously accomplished by Popova et al. (Figure 1, compound F),^22^ the 1:1 copolymerization of unfunctionalized PPXs diminishes the distinct effects of irregular elements. In contrast, this work eliminates such PPX intervention and presents a more in-depth investigation of the mechanical properties and oxidation treatment to form a uniform oxide layer, which are not explored in the reported works.

To probe the effects of such heavier element incorporation, trimethylgermane (GeMe_3_)- and trimethyltin (SnMe_3_)-substituted PPXs are deposited and analyzed. Elemental analysis (EA) and X-ray photoelectron spectroscopy (XPS) quantitatively revealed the surface oxidation of the polymer and the relative ratio of inserted oxygen.

## Result and Discussion

### Synthesis and Characterization of PPX-GeMe_3_ and PPX-SnMe_3_

The synthesis of the CVD precursors was commenced by the bromination of [2,2]­paracyclophane to obtain a 4,16-brominated compound, which yields the corresponding functionalized paracyclophane after lithiation and a nucleophilic substitution reaction (Figure 2a). Both GeMe_3_-cyclophane (GeMe_3_-cy, **1**) and SnMe_3_-cyclophane (SnMe_3_-cy, **2**) were successfully recrystallized from dichloromethane and hexane in moderate yields and high purities. The structures of the synthesized cyclophanes were confirmed by NMR spectroscopy (Figures S1–S4) and EA (Table S1). For the ^1^H NMR spectrum of **1**, the protons of the CH_2_–CH_2_ moiety in the cyclophane framework appear as two sets of multiplets, whereas in **2**, they appear as one consecutive multiplet. In the ^1^H NMR spectrum of **2**, the distinctive trimethyl signal accompanied by tin-satellite splitting was observed at around 0.34 ppm (Figure S3). Only germanium-functionalized **1** successfully underwent single-crystal X-ray diffraction (SC-XRD, Figure 2b), whereas multiple attempts to obtain a refined crystal structure for tin-functionalized **2** were unsuccessful. The crystal structure of **1** revealed the distance of Ge–Ph (Ge1–C16) as 1.980 Å and Ge–CH_3_ (Ge1–C17, −C18, and −C19) bonds to be 1.952, 1.952, and 1.957 Å, respectively. Overall, these lengths match the range reported in the literature.^27,28^ The distance of the CH_2_–CH_2_ bridging moiety of the cyclophane (C1–C2) is 1.585 Å, showing slightly shortened/elongated relative to PPX-N, which exhibits a distance of 1.579 Å.^29^

**Figure 2:**
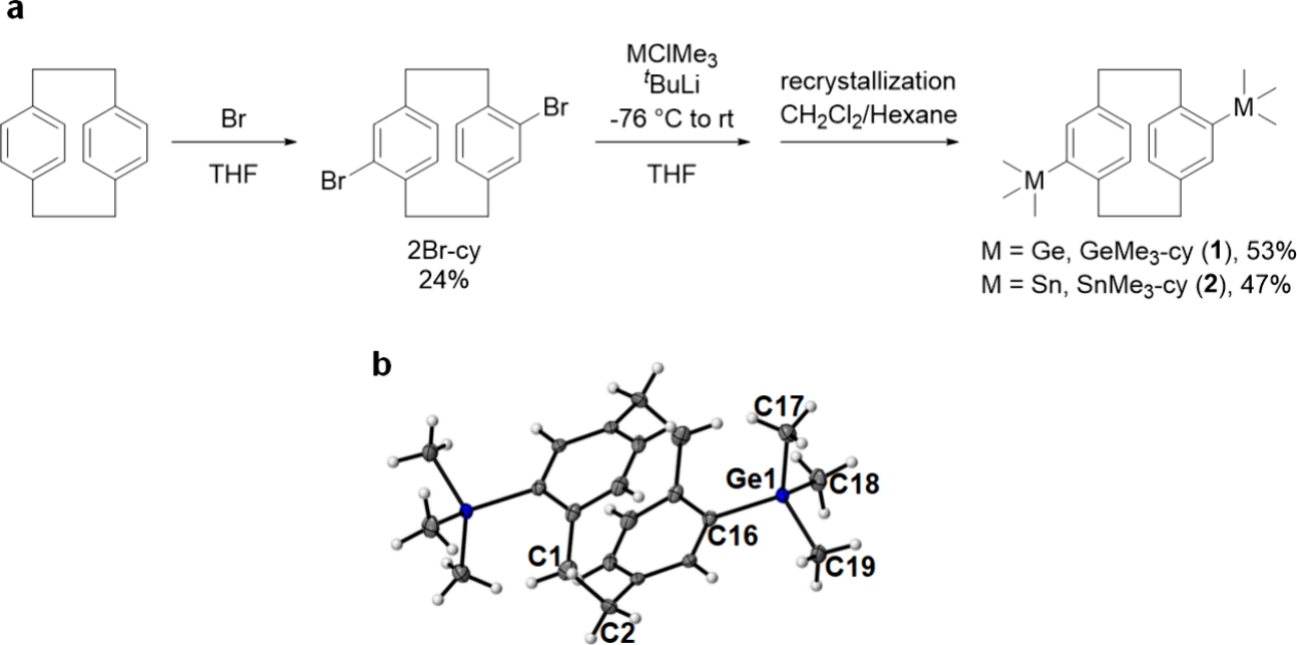
(a) Synthetic scheme for Ge- and Sn-substituted [2.2]­paracyclophanes **1** and **2**, respectively. (b) Oak Ridge thermal ellipsoid plot (ORTEP) drawing of **1**, which was obtained from single-crystal XRD data.

As for the CVD flow process, polymers are deposited through a linear quartz cylindrical chamber equipped with a Schlenk flask (Figure 3a): starting with vacuuming the chamber loaded with precursor, the pyrolysis zone is preheated to the corresponding pyrolysis temperature, and as the pressure is stabilized at 2.0–5.0 × 10^–2^ mbar, the sublimation zone is then heated to a specific temperature to initialize polymerization. Through several screenings, sublimation was optimized with a gradual temperature increase, starting with 170 °C and then slowly heating to 240 °C. A test tube was selected as the best container for loading the precursor to suppress “back draft”, a phenomenon that occurs to some dimers that are sublimed but never undergo pyrolysis even under an extremely high sublimation temperature and strong vacuum. As for the initial attempts, the expected polymers were not furnished under a pyrolysis temperature of 600–690 °C, which is common for commercial PPXs as well as PPX-SiMe_2_H.^23,30^ The resultant viscous oil indicated the decomposition at such a high temperature, possibly due to the dissociation of Ge– or Sn–Ph bonds. Another key feature is that polymerized PPX-GeMe_3_ and SnMe_3_ emerge at the deposition zone 1, which indicates that deposition temperatures for these PPXs are relatively higher than those of PPX-SiMe_2_H and other common PPXs. This observation excellently matches the theory of ceiling temperature, where the rates of depolymerization and polymerization are in equilibrium;^31^ the heavier and bulkier pyrolyzed intermediates generated from **1** and **2** are more prone to condense and deposit at a higher temperature than that of the less bulky precursor.

**Figure 3:**
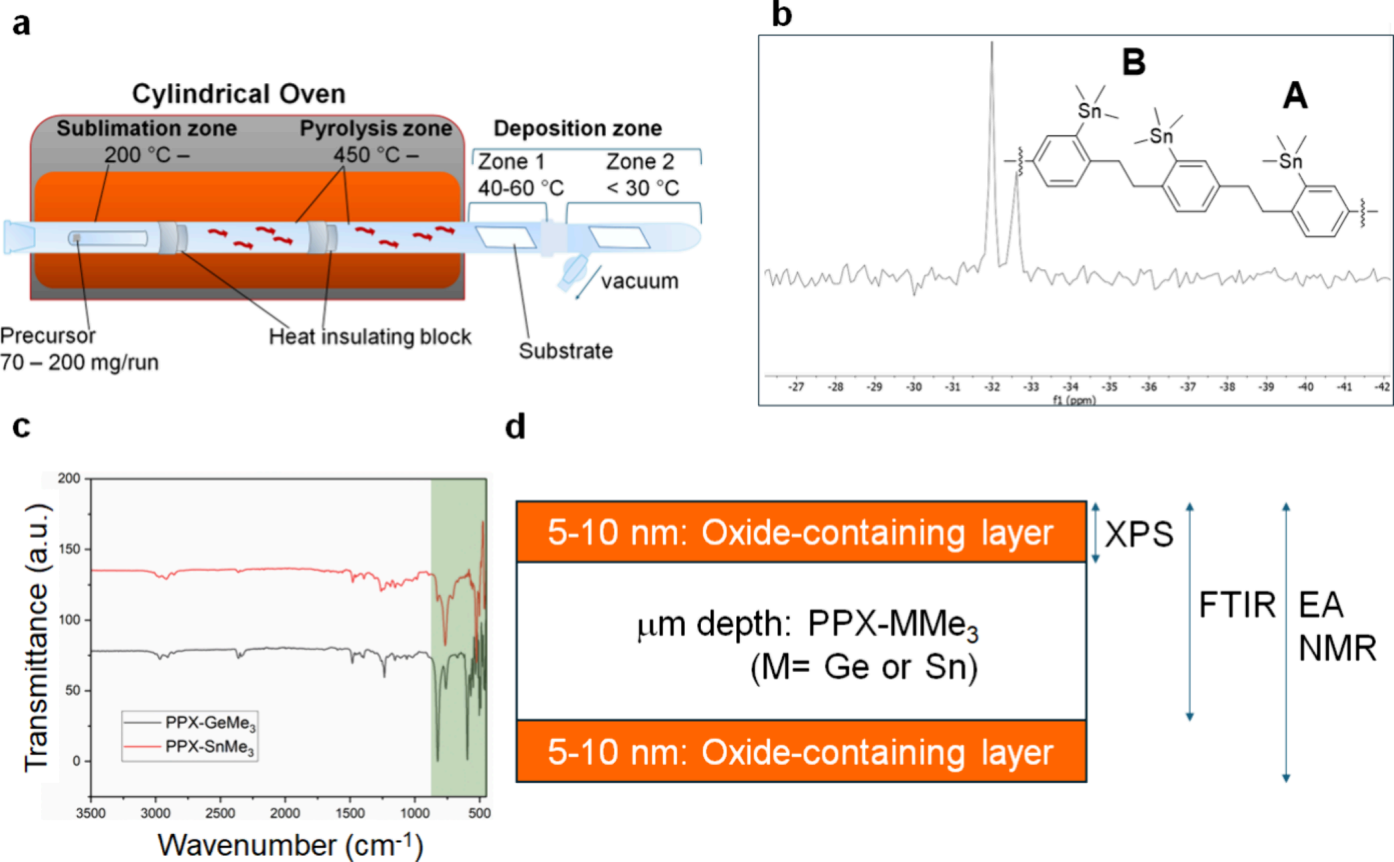
(a) Schematic diagram of the CVD system constructed with cylindrical quartz glass. (b) ^119^Sn-NMR spectrum of the PPX-SnMe_3_ film in CD_2_Cl_2_. (c) FTIR spectra of PPX-GeMe_3_ and PPX-SnMe_3_. (d) Cross-sectional diagram of the obtained PPXs specifying the depth of each measurement.

Through temperature screenings, the optimized pyrolysis temperatures of GeMe_3_-cy and SnMe_3_-cy were 450–560 and 430–480 °C, respectively. The absolute doublet-like signals at −31.99 and −32.62 ppm in the ^119^Sn-NMR spectrum (Figure 3b) are attributed to the unoxidized SnMe_3_ moieties with different linkage configurations (parts A and B, Figure 3b), which is also supported by the chemical shifts of the precursor **2** and simple Me_3_SnPh, emerging at −37.3 and −28.6 ppm, respectively.^30^ The signals indicate that tin oxide was not confirmed, as such oxides are usually insoluble in NMR solvents.

Regarding the green band shown in the FTIR spectra in Figure 3c, the absorption bands near ∼500 cm^–1^ are attributed to Ge-, Sn-phenyl stretching modes, while the signals around ∼900 cm^–1^ arise from Ge-, Sn-CH_3_ rocking vibrations. Additionally, the signals around 1500 cm^–1^ are assigned to C–C stretching in Ge- and Sn-substituted benzene rings. These vibrations are in good agreement with the literature.^22,32,33^ Although the signals could shift depending on the oxidation states, the range of Ge–O (or Sn–O) and O–Ge–O (O–Sn–O) vibration bands is typically observed in the lower region, ∼700 cm^–1^, usually centered around 600 cm^–1^.^34–36^ These regions may overlap with the Ge–, Sn–C, and C–C signals mentioned above; otherwise, the obtained spectra indicate the absence or minimal presence of oxygen-inserted units.

The chemical compositions and metal oxidation states of the deposited films in the surface region were determined by XPS and revealed the presence of the oxides of Ge and Sn. The Ge 2p spectrum acquired from PPX-GeMe_3_ (Figure 4a) shows two sharp signals at 1218.5 eV (Ge 2p_3/2_) with a full-width-at-half-maximum (fwhm) of 1.9 eV along with the spin–orbit pair peak at 1249.6 eV (Ge 2p_3/2_) (Δ*E* = 31.1 eV), demonstrating the presence of GeO.^37^ As for PPX-SnMe_3_ (Figure 4b), the Sn 3d core level indicates the presence of SnO through the sharp Sn 3d_5/2_ peak at 486.1 eV, with an fwhm of 1.6 eV, and a Sn 3d_3/2_ peak at 494.4 eV.^38^ The small shoulder at ≈496.5 eV might indicate the presence of another species or could be linked to an energy loss feature, albeit typically only observed in metals or at higher BEs; hence, the feature is not entirely clear.^39,40^ Nonetheless, the prevalent species is SnO, which can be supported by the characteristic shape of the valence band (Figure S11). Such metal oxidation^41^ is considered to occur through the cleavage of M–C bonds, and subsequent oxidation by the residual air remaining in the chamber.

**Figure 4:**
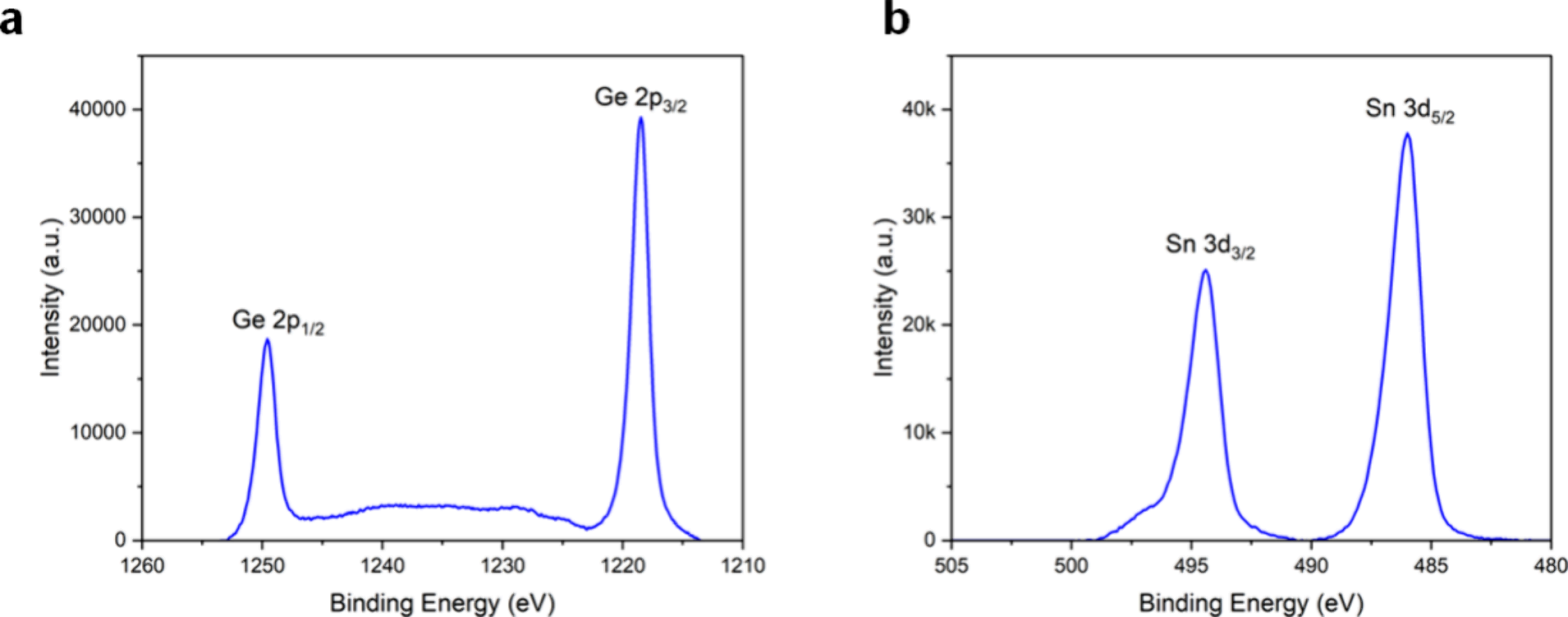
(a) XPS Ge 2p spectrum of the as-deposited PPX-GeMe_3_. (b) XPS Sn 3d spectrum of as-deposited PPX-SnMe_3_. A Shirley background was subtracted from the measured data.

The oxygen source for metal oxidation is considered to be either the residual air remaining in the cylindrical chamber or simply the atmospheric oxygen inserted after deposition to form the native oxides.

Figure 3d summarizes the interpretation that is concluded by the series of performed measurements and demonstrates the presence of GeO or SnO on the outermost surface of the films. The oxygen rates of as-deposited PPX-GeMe_3_ and PPX-SnMe_3_ are quantitatively determined by EA (Table S1) as 4.83 and 4.05 wt %, respectively. Overall, the oxide-incorporating layer is expected to be a few nanometers in depth in both novel PPXs.

### Physical Properties of PPX-GeMe_3_ and PPX-SnMe_3_

The mechanical properties revealed by the series of measurements are summarized in Table 1. TGA was performed to investigate and compare the thermal decomposition of films from this work with those of the known PPXs. The samples are heated from room temperature (25 °C) to 600 °C at a rate of 10 °C/min. According to the thermogravimetric curves, PPX-GeMe_3_ starts to lose mass as the temperature reaches 270–275 °C, whereas PPX-SnMe_3_ exhibits stepwise mass loss, first at 180–185 °C and then at 441 °C. Such volatilities indicate relative thermal instability compared to PPX-SiMe_2_H and PPX-SiMe_3_, which retain their mass up to 466–486 °C. Considering Ganguli et al.’s work,^42^ the rapid weight loss is typically attributed to the rupture of the shorter polymer chains. Although the exact mechanism is still unknown in detail, the relatively bulky substituent (GeMe_3_ or SnMe_3_, in our case) on the carbon adjacent to the reacting site may interrupt the growth of the activated quinone-type intermediate by steric hindrance and result in a bulk of short polymer chains. As such, it is plausible to suggest that the polymers with larger and highly cross-linked chains, like PPX-SiMe_2_H, would be more thermally durable. In this respect, Ganguli et al.^42^ additionally mentioned that a lower deposition temperature improves thermal stability, which matches the observed high deposition temperature of PPX-GeMe_3_ and -SnMe_3_. Comparing the TGA results of a similar Ge-containing PPX reported by Popova et al. (Figure 1, compound F-Ge),^22^ which exhibits a decomposition temperature of approximately 300 °C, the presence of Ge in every polymer unit in PPX-GeMe_3_ does not significantly affect the thermal properties. Since the compound F–Ge is a copolymer with unfunctionalized PPXs and the cross-linked moieties are not evenly distributed, it is less rational to directly compare the thermal stability of PPX-GeMe_3_ and compound F–Ge concerning the cross-linking effect.

**Table 1:** Mechanical Properties of Modified PPX Films

PPX	Young’s modulus (GPa)	percentage of elongation (%)	contact angle (°)	decomposition temperature (°C)
PPX-GeMe_3_	1.34 ± 0.28	11–23	90	272
PPX-SnMe_3_	5.30 ± 0.41	6–16	96	185
PPX-Cl^a^	3.44 ± 0.29	20–30	85	442
PPX-SiMe_3_ ^a^	2.86 ± 0.31	2–10	95	466
PPX-SiMe_2_H^a^	1.18 ± 0.24	60–90	99	486

a Deposited with SCS Labcoter 2 (PDS 2010).

The toughness and ductility of PPX-GeMe_3_ and PPX-SnMe_3_ are compared with those of PPXs deposited using an SCS Labcoter 2 PDS (2010) system, which features continuous chamber pressure monitoring and control: as for the deposition of PPX-Cl, -SiMe_3_, and -SiMe_2_H, the pressure is kept at 2.0 × 10^–2^ mbar, sublimation and pyrolysis are performed at 170 and 690 °C, respectively. As previously mentioned, in the deposition system shown in Figure 2a, the sublimation, pyrolysis, and deposition temperatures are optimized for Ge- and Sn-PPX. Meanwhile, the pressure is not held constant; however, it fluctuates within 0.3–0.5 × 10^–2^ mbar of the initial pressure, which ranges from 4.0 to 4.5 × 10^–2^ mbar.

PPX-GeMe_3_ revealed less than half of Young’s modulus as obtained in PPX-Cl, indicating the enhancement of softness and similar elasticity as that of PPX-SiMe_2_H (Figure S12). As PPX-SnMe_3_ exhibited a higher Young’s modulus compared to its lighter analogues, the incorporation of Sn resulted in higher robustness, which enables it to resist deformation under higher stress. Both of our PPXs overcome the elongation percentage of their Si-analogue PPX-SiMe_3_. Overall, the novel PPXs preserve the ductility of the PPX-Cl film and exhibit slightly enhanced hydrophobicity (Table 1), regardless of the structurally involved germanium and tin.

### Oxygen Plasma Treatment of PPX-GeMe_3_ and PPX-SnMe_3_ and Confirmation of Surface Passivation

Oxygen plasma surface treatment and sodium hypochlorite (NaOCl) oxidation were performed for 8 min and 65 h, respectively. As the entire spectra are not significantly changed despite the noise at the lower wavenumbers (Figure 5a,b), both plasma- and chemical-mediated methods made minimal impact on the inner oxygen rate. Cross-linking, more specifically, the expansion of the chain network, suppresses rupture and enhances thermal stability, as discussed in the previous section. In support of this, the TGA spectra (Figure 5c,d) show that the patterns of mass loss remain largely preserved after the treatments. This indicates that the majority of the polymer chains remain noncross-linked, evidenced by the absence of a notable shift in degradation temperature, despite slight variations in the mass loss gradient. The ^119^Sn-NMR spectra further indicate that the main structure of the PPX-SnMe_3_ is retained intact after treatment, as no significant chemical shift, intensity variation, or emergence of new signals is observed. (Figure 5e) Such the formation of an outer oxide-mixed crust could be applicable to the field of semiconductors, namely, as the metal-oxide-semiconductor (MOS) capacitor.^42,43^ This trend of the preserved thermal stability across the treatments is also observed for PPX-Cl and PPX-SiMe_2_H (Figures S13 and S14).

**Figure 5:**
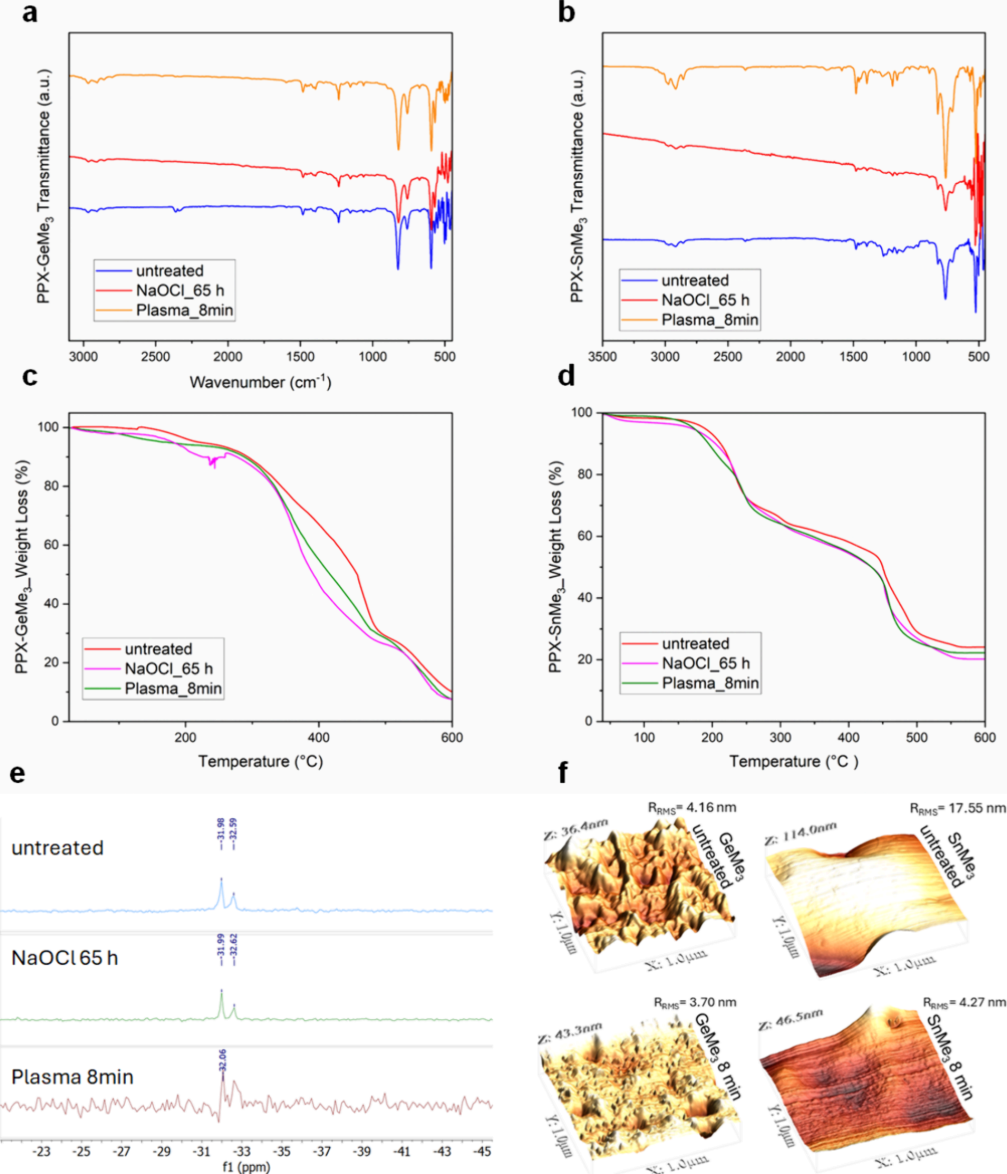
(a) FTIR spectra of untreated, O_2_ plasma, and NaOCl-treated PPX-GeMe_3_. (b) FTIR spectra of untreated, O_2_ plasma, and NaOCl-treated PPX-SnMe_3_. (c) TGA of untreated, O_2_ plasma, and NaOCl-treated PPX-GeMe_3_. (d) TGA of untreated, O_2_ plasma, and NaOCl-treated PPX-SnMe_3_. (e) ^119^Sn-NMR spectra of untreated, plasma, and NaOCl-treated PPX-SnMe_3_. (f) AFM images of untreated and plasma treated, left: PPX-GeMe_3_ and right: PPX-SnMe_3_.

The surface topographies of the as-deposited and plasma-treated films are determined by atomic force microscopy (AFM), as shown in Figure 5f. The surface roughness values *(R*_RMS_) of both PPXs decreased, indicating that polishing has occurred through plasma treatment. This also supports the presence of an already formed oxide-rich surface layer, as GeO and SnO have dense topographies, and in such a case, plasma acts more like a polishing agent and uniformly removes the larger asperities on the surface. This is explained by oxygen ion bombardment and de-excitation of the metastable species, and such a phenomenon is relatively uncommon in material plasma treatment, yet has been reported in previous studies.^44–46^

Finally, the surface chemical compositions of the plasma-treated PPXs are evaluated by XPS measurement (Figure 6). In the Ge 2p spectrum acquired from PPX-GeMe_3_, the binding energy (BE) of Ge 2p_3/2_ is located at 1220.9 eV (fwhm = 2.3 eV), which is shifted to higher BEs from the 2p_3/2_ signal of the as-deposited version by 2.4 eV. While the BE indicates a higher degree of oxidation linked to GeO_2_, the difference between Ge 2p_3/2_ and Ge 2p_1/2_ remains unchanged at 31.1 eV.^47,48^ However, the change in oxidation state can be confirmed upon comparison of the valence band structure.^49^ As for the PPX-SnMe_3_ polymer, the Sn core level reveals one species, with a BE of the Sn 3d_5/2_ signal at 487.1 eV (fwhm = 1.7 eV) together with its spin–orbit pair at 495.6 eV (Δ*E*= 8.5 eV). This BE, alongside the valence spectra, is in good agreement with the literature on SnO_2_.^50,51^

**Figure 6:**
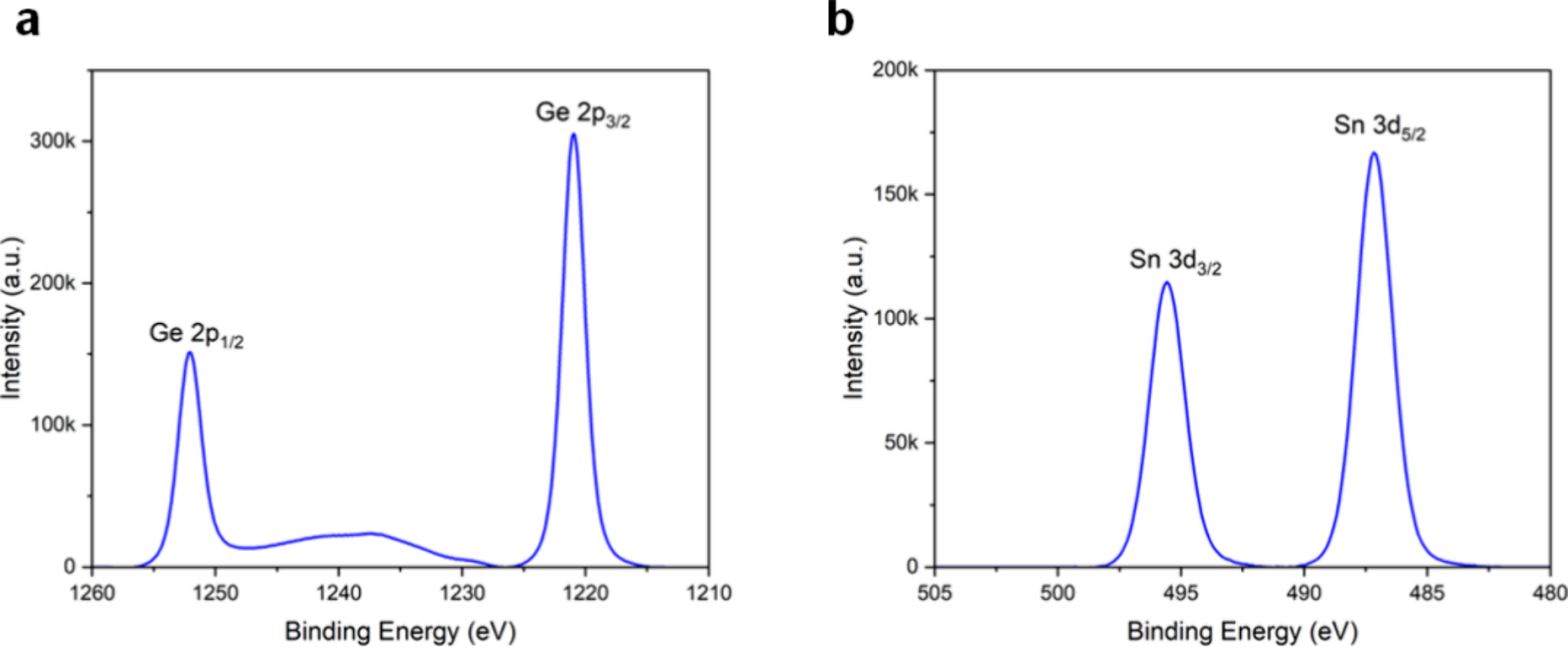
(a) XPS Ge 2p spectrum of plasma-treated PPX-GeMe_3_. (b) XPS Sn 3d spectrum of plasma-treated PPX-SnMe_3_. A Shirley background was subtracted from the measured data.

## Conclusions

Ge- or Sn-incorporated paracyclophane precursors were prepared through a simple lithiation reaction and facile purification. Through the CVD process, the polymerization of self-standing, transparent organic polymers incorporating Ge and Sn and their oxide units are accomplished. The obtained PPX films exhibited mechanical properties, with one being soft and the other stiff, while both retained the ductility of PPX-Cl. The results from oxygen plasma treatment and chemical oxidation demonstrated the effective surface passivation and preservation of the inner core of both PPX-SnMe_3_ and PPX-GeMe_3_ films.

Given that the examples of organic polymers incorporating Ge and Sn are limited to date, this work serves as a benchmark for such a unique polymer as well as providing insights into new designs of transparent coating materials. The novel films could further be applied as MOS capacitors since the polymerizing procedure provides excellent transparency and an oxide-rich layer with nanometer-order thickness. Subsequent investigations regarding the MOS application will soon be conducted.

## Experimental Section

### Materials and Methods

[2.2]­Paracyclophane was purchased from BLD Pharm. Chlorotrimethylgermane, trimethyltin chloride, and *tert-b*utyllithium solutions (1.7 M, pentane) were purchased from Sigma-Aldrich. Tetrahydrofuran (THF) was used for the synthesis of the precursors 4,16-bis­(trimethylgermanyl)[2.2]­paracyclophane (**1**) and 4,16-bis­(trimethylstannyl)[2.2]­paracyclophane (**2**) and was dried before use. The synthesis and purification of **1** and **2** were conducted under an argon atmosphere.

### Synthesis of 4,16-Dibromo[2.2]­paracyclophane

[2.2]­Paracyclophane (50 g, 240.03 mmol, 1 equiv) was suspended in 800 mL of chloroform. After the quick addition of 80 mL of bromine, the solution was stirred under reflux for 3 h. After cooling the reaction mixture to 0 °C, a saturated NaHSO_3_ solution was added dropwise until the reaction mixture discolored. The precipitate was filtered and washed 3 times with 10 mL of water and 2 times with 10 mL of ethanol.

^1^H NMR (400 MHz, C_6_D_6_) δ (ppm) = 7.06 (dd, *J* = 7.8, 1.8 Hz, 2H), 6.29 (d, *J* = 1.8 Hz, 2H), 6.05 (d, *J* = 7.8 Hz, 2H), 3.30 (ddd, *J* = 13.0, 10.4, 2.2 Hz, 2H), 2.85 (ddd, *J* = 12.6, 10.4, 4.8 Hz, 2H), 2.57–1.77 (m, 4H); ^13^C NMR (101 MHz, CDCl_3_) δ (ppm) = 141.19 (s), 138.56 (s), 134.15 (s), 128.29 (s), 126.76 (s), 35.39 (s), 32.85 (s).

### Synthesis of 4,16-Bis­(trimethylgermanyl)[2.2]­paracyclophane (**1**)

A portion (1.0 g, 2.73 mmol, 1.0 equiv) of dibromo[2.2]­paracyclophane was dissolved in 100 mL of tetrahydrofuran (THF). After cooling the solution to −76 °C, 6.59 mL (11.20 mmol, 4.1 equiv) of a 1.7 M solution of *t*-BuLi was added over 20 min. The reaction mixture was stirred for 45 min at −76 °C and then warmed up to 0 °C. After reaching 0 °C, the reaction mixture was cooled back down again to −76 °C, and 1.38 mL (11.20 mmol, 4.1 equiv) of chlorotrimethylgermane was added over 10 min. The reaction mixture was stirred for 16 h and allowed to warm to room temperature during that time. The solvent was removed, and dichloromethane (15 mL) was added and then filtered. The resulting filtrate was put into a −26 °C freezer and recrystallized. White needle-like crystal was obtained after the removal of the solvent (yield = 53%).

^1^H NMR (400 MHz, C_6_D_6_) δ 6.75 (s, 2H), 6.37 (d, *J* = 9.8 Hz, 2H), 6.23 (d, *J* = 7.8 Hz, 2H), 3.03–2.96 (m, 6H), 2.87–2.75 (m, 2H), 0.44 (s, 18H); ^13^C NMR (101 MHz, C_6_D_6_) δ 145.13, 141.57, 138.21, 136.63, 134.32, 133.75, 36.07, 35.86, 0.18.

### Synthesis of 4,16-Bis­(trimethylstannyl)[2.2]­paracyclophane

Dibromo­[2.2]­paracyclophane (4.0 g, 10.93 mmol, 1.0 equiv) was dissolved in 150 mL of THF. After cooling the solution to −76 °C, 28.28 mL (48.07 mmol, 4.4 equiv) of a 1.7 M solution of *t*-BuLi was added over 30 min. The reaction mixture was stirred for 45 min at −76 °C, and 9.58 g (48.07 mmol, 4.4 equiv) of trimethyltin chloride dissolved in 5 mL of THF was added over 10 min. The reaction mixture was warmed up to room temperature and stirred for 16 h. After removal of the solvent and recrystallization in dichloromethane/hexane at −26 °C, the desired product was obtained as a shiny white crystal (yield = 47%).

^1^H NMR (400 MHz, C_6_D_6_) δ 6.79 (s, 2H), 6.45–6.22 (m, 4H), 3.10–2.85 (m, 8H), 0.34 (s, 18H); ^13^C NMR (101 MHz, C_6_D_6_) δ 146.75, 144.14, 139.09, 138.15, 134.62, 132.98, 38.16, 36.03, −8.20; ^119^Sn-NMR (80 MHz, C_6_D_6_) δ (ppm) = −37.06 (s).

### Chemical Vapor Deposition

To begin with, substrates (e.g., silicon wafers and simple glass plates) were put into the deposition zone of the tube chamber. The corresponding amount of the precursor, which was wrapped in aluminum foil and placed in a test tube, was introduced into the sublimation zone. This chamber was capped with a ground-glass stopper and a Schlenk flask and then transferred into the CARBOLITE GERO coating device. The entire chamber was evacuated, and the pyrolysis zone was preheated to the corresponding temperature. Once this temperature was reached, the sublimation zone was heated to the desired temperature. Transparent PPX films were obtained in the deposition zone, and the whole polymerization process was typically conducted within 10 min.

### Oxygen Plasma Treatment

Diener Electronics’ low-pressure plasma system “FEMTO” was used to treat the obtained film with oxygen plasma. First, the entire chamber was flushed with oxygen 4 times. Subsequently, the films were placed, the plasma chamber was set to 60 W, and an O_2_ gas with a flow of 20 mL min^–1^ was set.

### Chemical Treatment

For the chemical treatment postpolymerization, aquatic NaOCl (6–14% active chlorine) was used. After immersing the polymer films for 65 h, the films were washed multiple times with H_2_O and dried in a vacuum prior to analysis.

### Atomic Force Microscopy (AFM)

AFM images were recorded with a Dimension ICON by Bruker in tapping mode. All measured samples were deposited on silicon wafers. The images were created and analyzed toward the root-mean-square roughness (RMS) with the scanning probe microscopy software WSxM (Version 5.0).^52^

### Attenuated Total Reflection Fourier-Transform Infrared (ATR-FTIR)

ATR-FTIR spectra were measured on a Bruker Vertex70v ATR-FTIR spectrometer. Here, the detector was cooled with liquid nitrogen and a background measurement was performed prior to every measurement.

### Differential Scanning Calorimetry (DSC)

Differential scanning calorimetry analysis was carried out on a DSC Q2000. Here, roughly 2–4 mg of previously dried functionalized PPX was sealed under air in a DSC aluminum pan. DSC analyses were conducted from 0 °C to a corresponding temperature with a heating rate of 10 °C/min.

### Nuclear Magnetic Resonance Spectroscopy (NMR)

To obtain NMR spectra, a Bruker Avance Neo 400 MHz or Avance 500 MHz spectrometer was used. The evaluation of the spectra was performed by using MestReNova (version 15.0.0). The following abbreviations are used for the different multiplicities of NMR spectra obtained: s = singlet, d = doublet, dd = doublet of doublet, ddd = doublet of doublet of doublet, t = triplet, hept = heptet, and m = multiplet.

### Single-Crystal X-ray Diffraction Analysis

The Single-crystal X-ray intensity data were collected on an X-ray single-crystal diffractometer equipped with an X-ray single-crystal diffractometer with a CMOS detector (Bruker Photon-100), an IMS micro source with MoKα radiation (λ = 0.71073 Å), and a Helios mirror optic. An APEX4 software package was used for the measurement.^53^ The measurement was performed on single crystals coated with perfluorinated ether. The crystal was fixed on top of a microsample and measured under a stream of cold nitrogen (100 K). A matrix scan was used to determine the initial lattice parameters. Reflections were merged and corrected for Lorenz and polarization effects, scan speed, and background using SAINT.^54^ Absorption corrections including odd- and even-ordered spherical harmonics were performed using SADABS.^55^ Space group assignments were based on systematic absences, E statistics, and successful refinement of the structures. Structures were solved by direct methods with the aid of successive difference Fourier maps and were refined against all data using the APEX4 ^53^ in conjunction with SHELXL-2018/3.^56,57^ and SHELXLE.^58^ Methyl hydrogen atoms were refined as part of rigid rotating groups, with a C–H distance of 0.98 Å and Uiso­(H) = 1.5·Ueq­(C).

Other H atoms were placed in calculated positions and refined using a riding model, with methylene and aromatic C–H distances of 0.99 and 0.95 Å, respectively, and Uiso­(H) = 1.2·Ueq­(C). Full-matrix least-squares refinements were carried out by minimizing Δ*w*(Fo^2^-Fc^2^)^2^ with a SHELXL-97 ^59^ weighting scheme. Neutral atom scattering factors for all atoms and anomalous dispersion corrections for the nonhydrogen atoms were taken from International Tables for Crystallography.^60^ Images of the crystal structures were generated by PLATON and MERCURY.^61,62^

The CCDC number 2491478 contains the supplementary crystallographic data for the structures 4,16-bis­(trimethylgermanyl)[2.2]­paracyclophane (**1**). These data can be obtained free of charge from the Cambridge Crystallographic Data Centre via https://www.ccdc.cam.ac.uk/structures/. The CIF file was generated using FinalCif.^63^

### Tensile Testing

For the mechanical characterization, a tensile tester (Universal Testing Machine, TesT GmbH, Germany) with a 50 N load cell and rubber clamp holders was used with the software TesTWinner 950 (TesT GmbH, Germany). The samples were stretched with a constant velocity of 10 mm·min^–1^ until breakage. The force was measured against the displacement of the initial length.

### Thermogravimetric Analysis (TGA)

The TGA was conducted on a “TG 209 F1 Libra” from NETSCH. All measurements were conducted under an argon atmosphere, and a sample mass of 1–2 mg was used. The heating rate was 10 °C/min, and a maximum temperature of 800 °C was chosen.

### Thickness Measurement

The thicknesses of PPX films were obtained through a 3D laser scanning confocal microscope (VK-X200, Keyence, Japan).

### X-ray Photoelectron Spectroscopy (XPS)

To characterize the surface changes, X-ray photoelectron spectroscopy (XPS) measurements were performed. For that, the samples were mounted onto a stainless-steel holder using an adhesive copper tape (PPI Adhesive Products, Ireland), transferred into the spectrometer (Axis Supra, Kratos, UK), and evacuated to a pressure below 10^–8^ Torr. All spectra were recorded using monochromatized Al–K_α_ radiation (1486.6 eV) and an emission current of 10 mA. The analyzer was operated at a pass energy of 40 eV for the core-level spectra and at 160 eV for the survey scan.

Ge 2p was investigated in the binding-energy (BE) range between 1260 and 1210 eV using 5 sweeps with a step size of 0.1 eV and a dwell time of 1000 ms, and Sn 3d was investigated in the BE range of 505 and 475 eV using five sweeps with a step size of 0.1 eV and a dwell time of 400 ms. The survey scan was performed using a BE range from 1200 to 5 eV, with two sweeps, a step size of 1.0 eV, and a dwell time of 100 ms. For energy corrections, the C 1s spectrum was also recorded in the BE range of 305–272 eV, with a step size of 0.1 eV and a dwell time of 400 ms. The energy scale of the spectra was then corrected by using the adventitious carbon peak at a BE of 284.8 eV. Then, a Shirley background was subtracted and a fit function with a Gaussian–Lorentzian 30% blend was used to analyze the spectra. For data analysis, CasaXPS, Version 2.3.24, Casa Software Ltd., UK, was used.

## Supplementary Material



## Notes

^1^H NMR spectra of compounds **1** and **2**, untreated and treated PPXs, ^13^C NMR spectra of compounds **1** and **2**, DSC spectra of untreated polymers, crystal data and structure refinement for compound **1**, unrefined crystal structure of **2**, additional XPS spectra, tensile testing data, TGA spectra of known PPXs, table of elemental analysis, and photograph of the polymerization set up (PDF)

## Abbreviations

NMR: nuclear magnetic resonance spectroscopy
FTIR: Fourier-transform Infrared spectroscopy
TGA: thermogravimetric analysis
MOS: metal-oxide semiconductor
