# Supplementary material for: Synthesis, Characterization, and Polymerization of Ge- and Sn-Substituted [2.2]Paracyclophanes toward Poly(para-xylylene) Films and Their Mechanical Properties
Source: Inorg Chem. 2026 Jan 30;65(6):3396–406. doi: 10.1021/acs.inorgchem.5c04945 (PMC12914628; doi:10.1021/acs.inorgchem.5c04945)
Supplement: Supplementary file 1 [file ic5c04945_si_001.pdf]

Supporting Information

Synthesis, Characterization, and Polymerization of Ge- and Sn-Substituted [2.2]Paracyclophanes toward Poly(para-xylylene) Films and Their Mechanical Properties

Moena Hirao, Lukas Bichlmaier, Tetsuhiko F. Teshima, Rebecca Wilhelm, Shigeyoshi Inoue*

[1] Technical University of Munich, TUM School of Natural Sciences, Department of Chemistry, Catalysis Research Center and Institute of Silicon Chemistry, Lichtenbergstraße 4, 85748 Garching, Germany

[2] Medical & Health Informatics Laboratories NTT Research Incorporated, 940 Stewart Dr., Sunnyvale, CA 94085, USA

[3] Technical University of Munich, TUM School of Natural Sciences, Department of Chemistry and Catalysis Research Center, Chair of Technical Electrochemistry, Lichtenbergstraße 4, 85748 Garching, Germany

*Corresponding author: s.inoue@tum.de

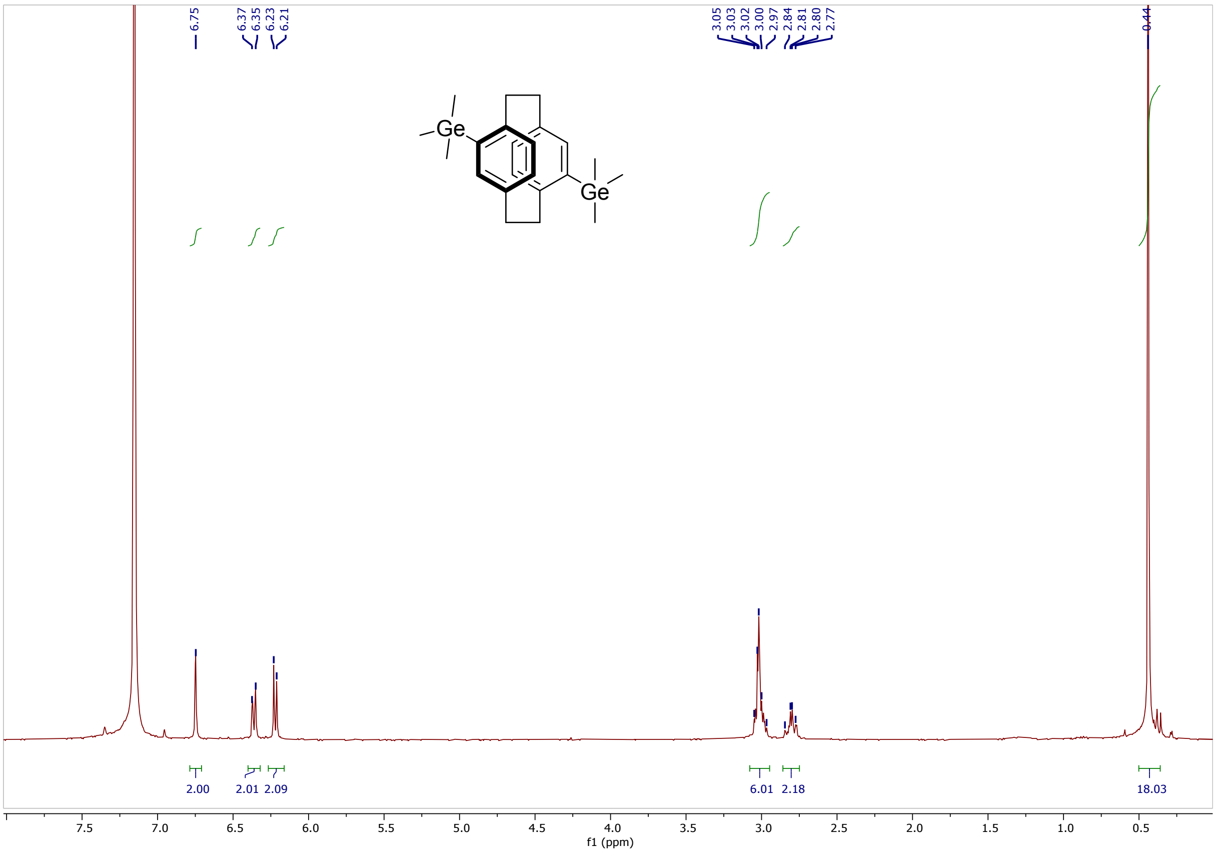

Figure S1. ^{1}H NMR spectrum of **1**.

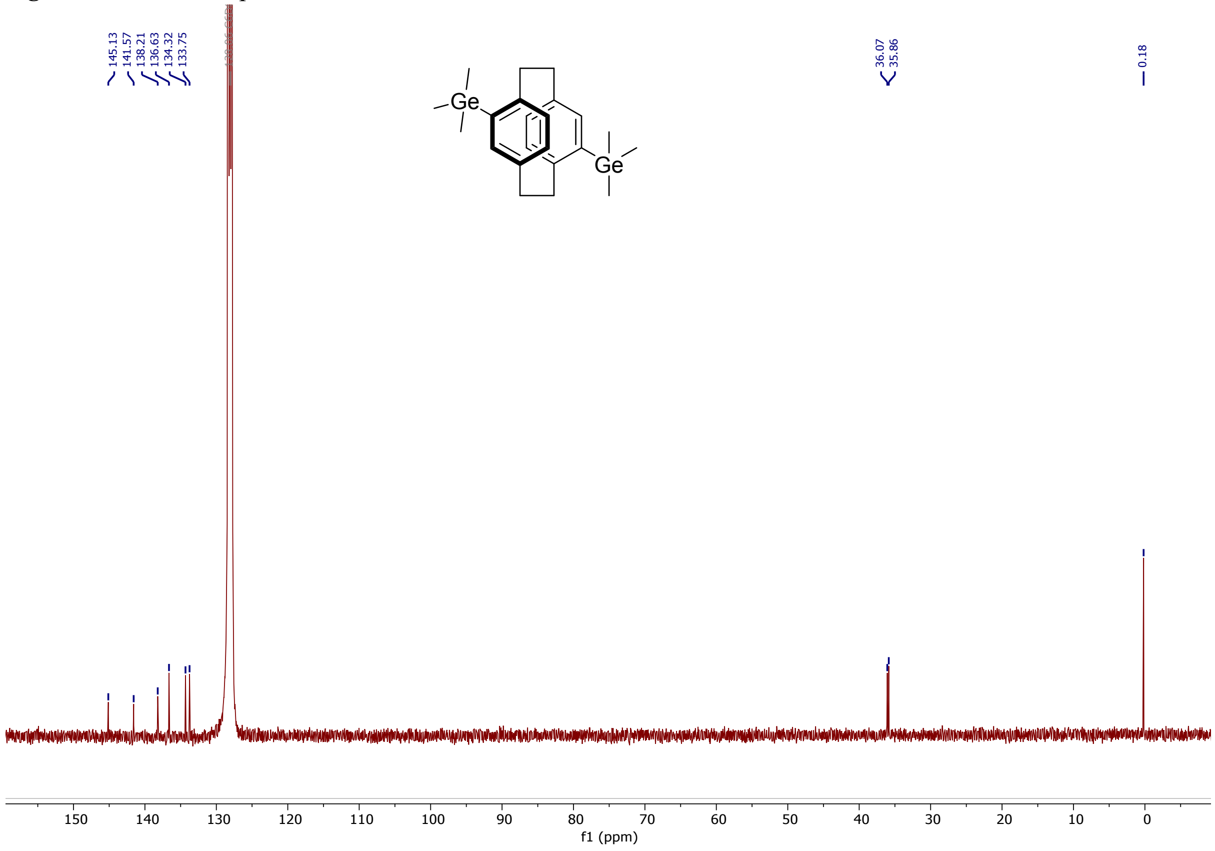

Figure S2. ^{13}C NMR spectrum of **1**.

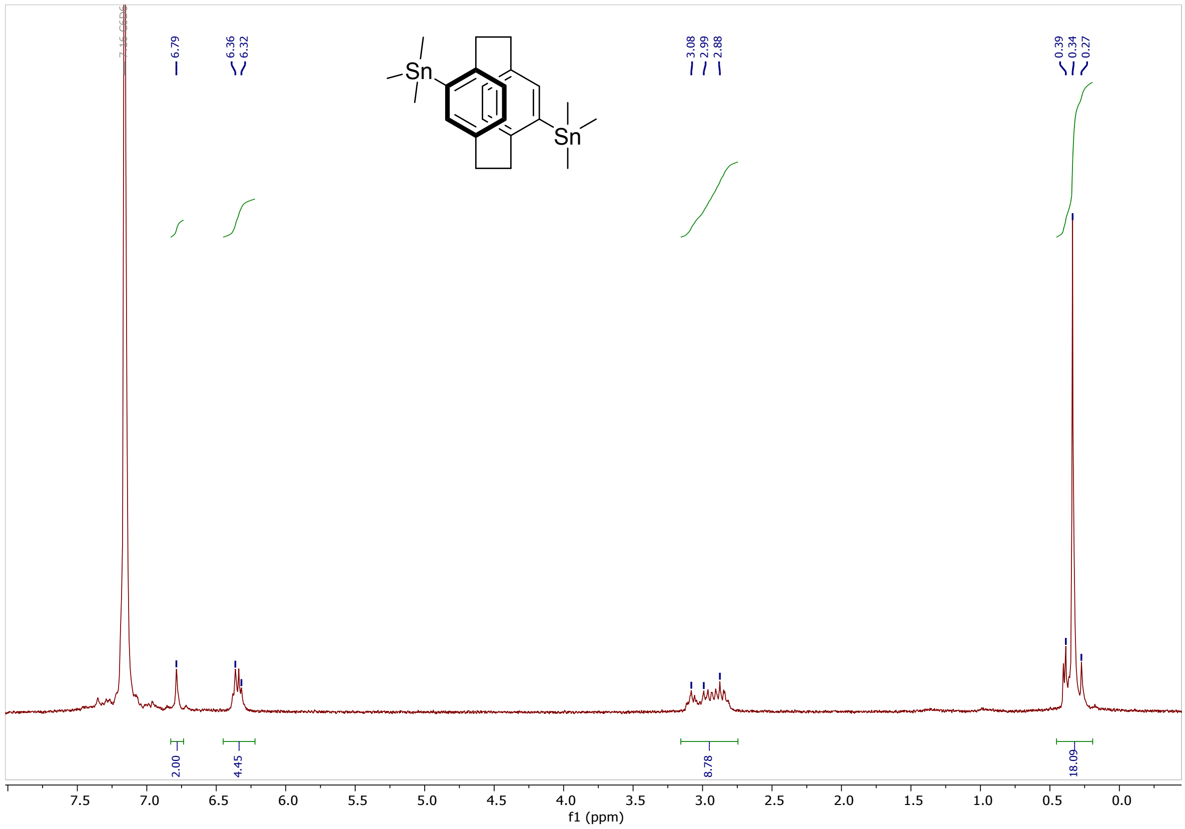

Figure S3. ^{1}H NMR spectrum of **2**.

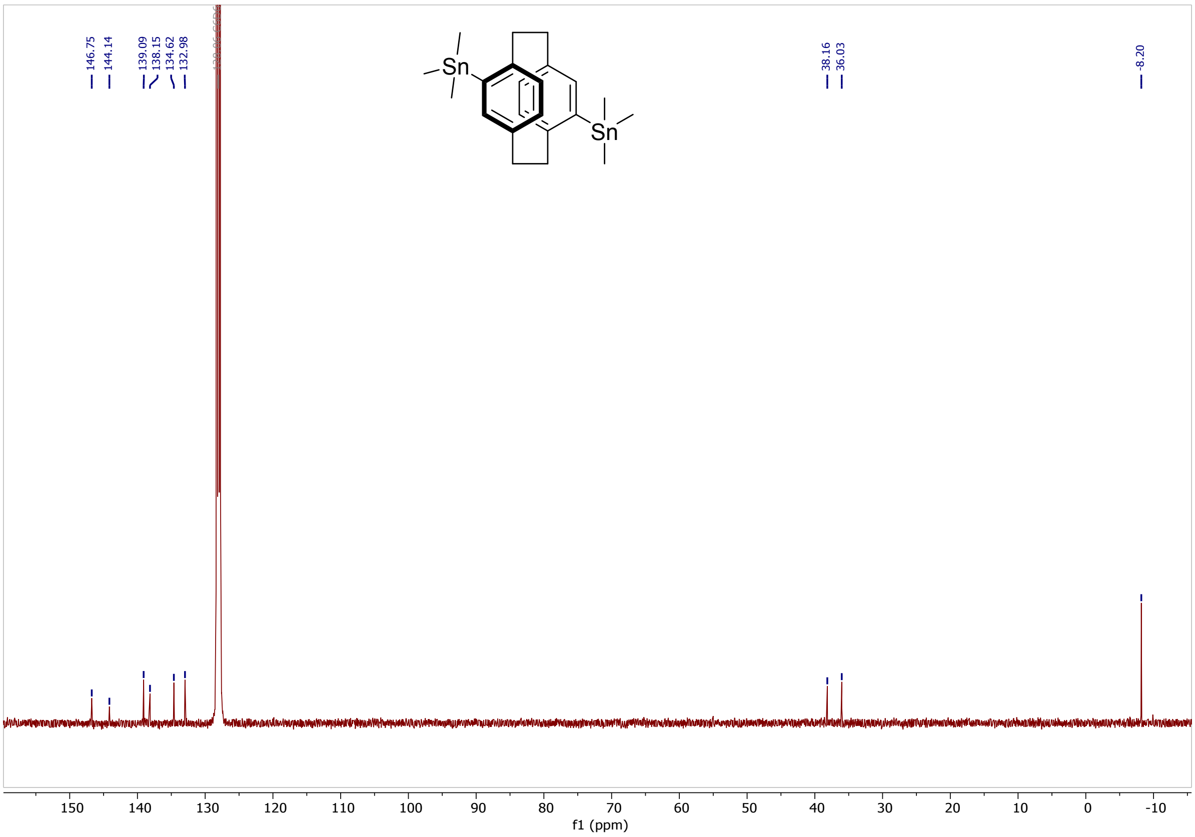

Figure S4. ^{13}C NMR spectrum of **2**.

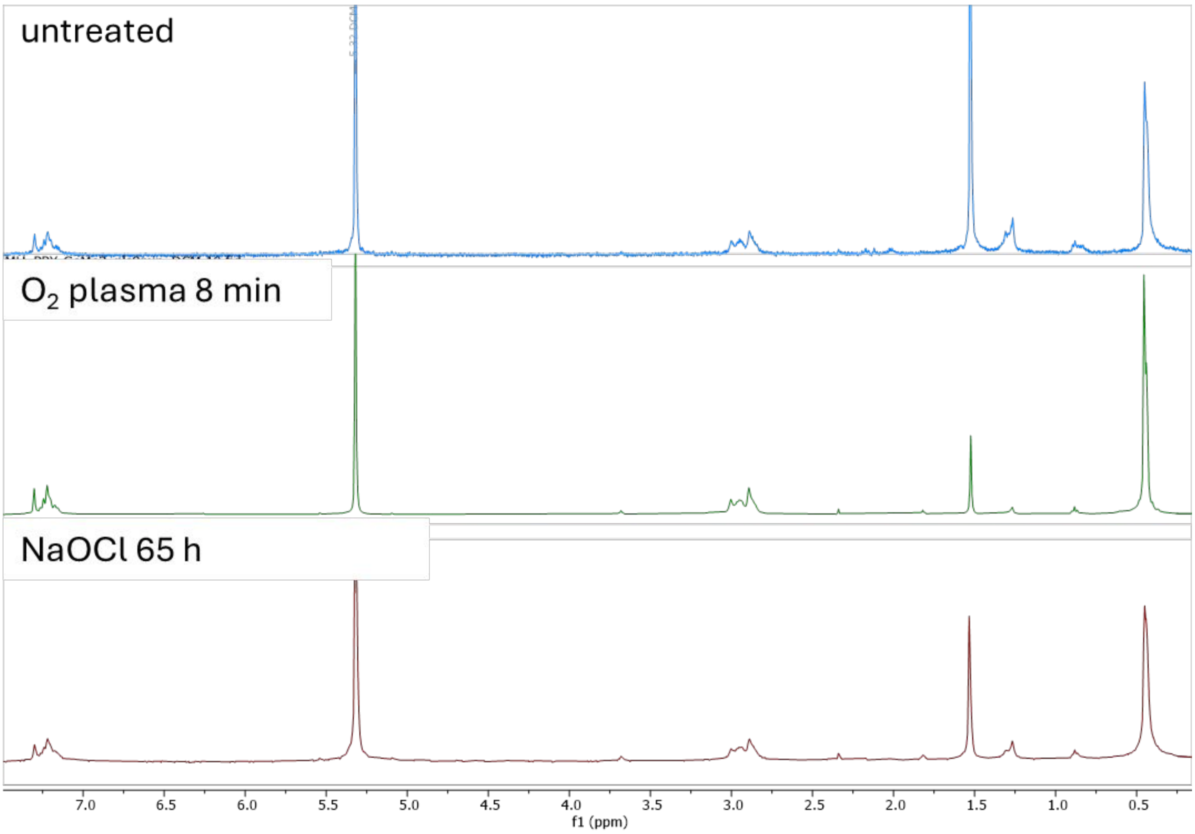

Figure S5. Stacked ^{1}H NMR spectra of as-deposited, plasma-treated, and NaOCl-treated PPX-$GeMe_3$.

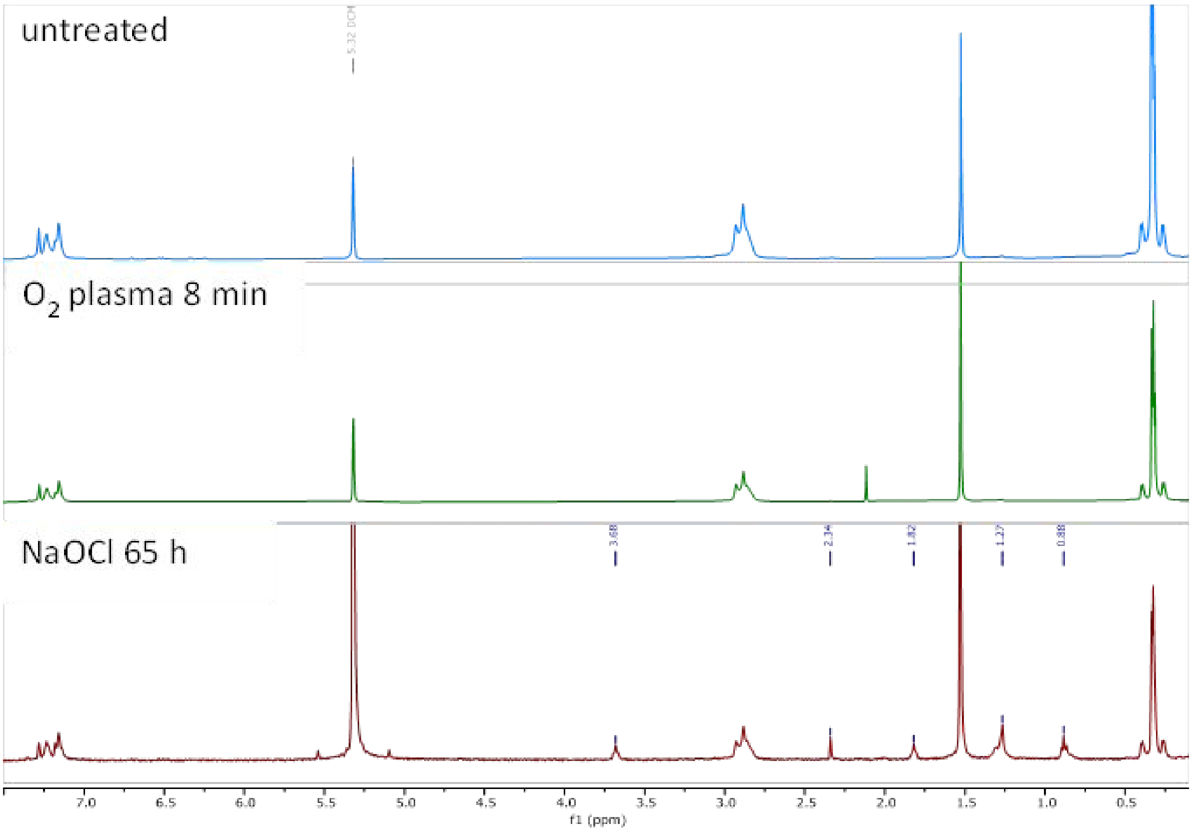

Figure S6. Stacked ^{1}H NMR spectra of as-deposited, plasma-treated, and NaOCl-treated PPX-$SnMe_3$.

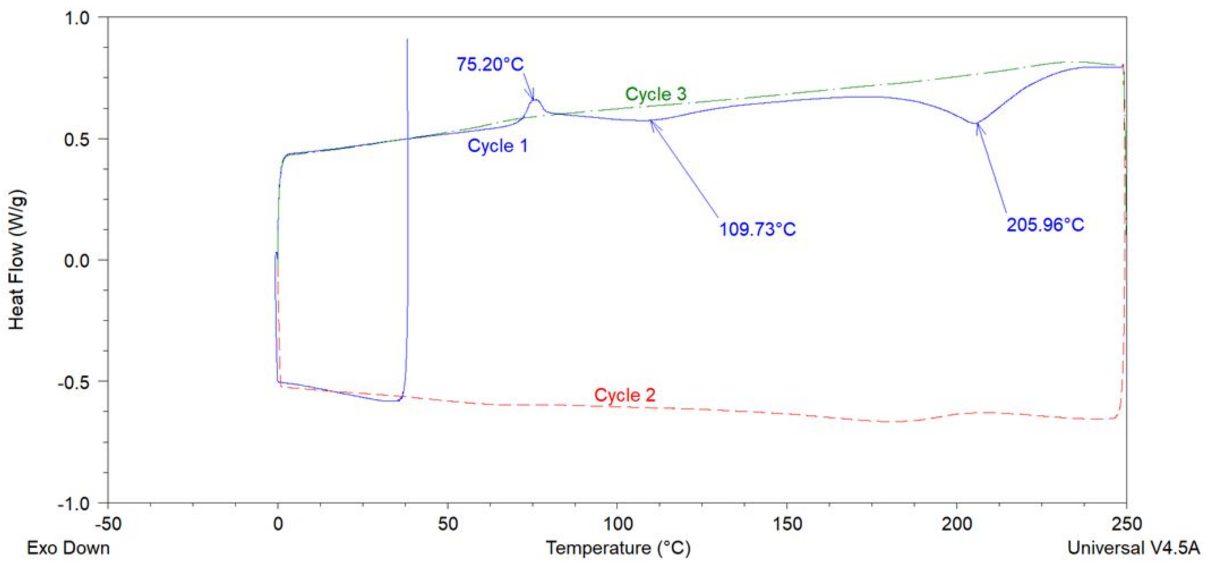

Figure S7. DSC curve of PPX-$GeMe_3$.

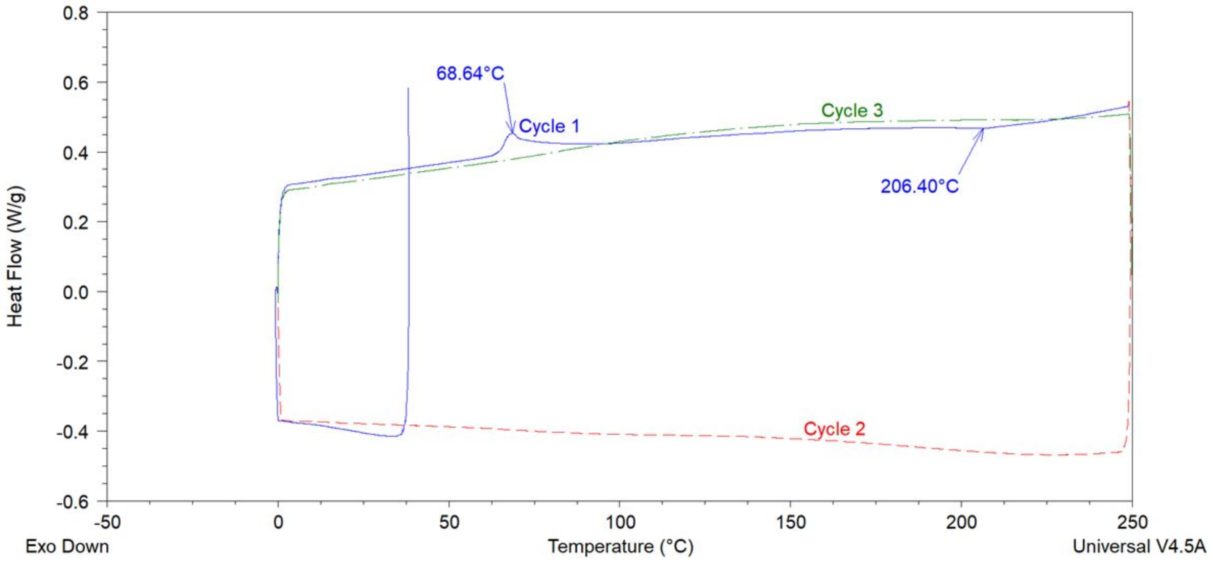

Figure S8. DSC curve of PPX-$SnMe_3$.

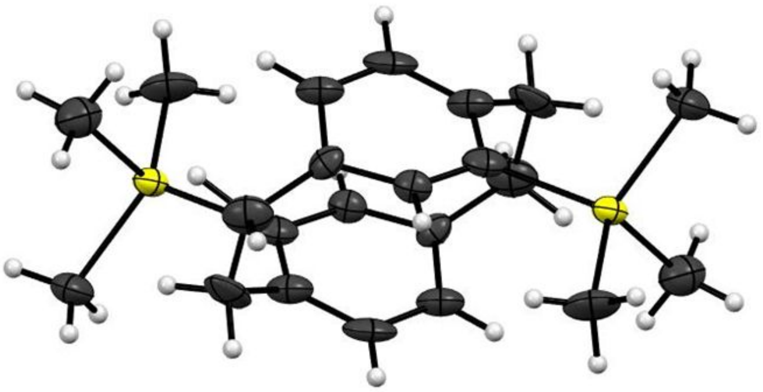

Figure S9. Unrefined ORTEP diagram of $SnMe_3$-cy (**2**), where yellow atoms are tin, gray atoms are carbon, and others are hydrogens.

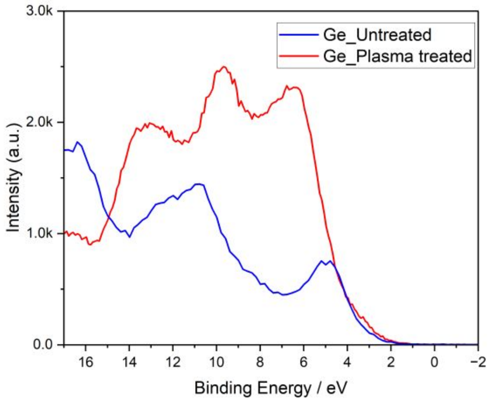

Figure S10. The valence band acquired from the plasma-treated PPX-$GeMe_3$. The spectrum pattern matches the literature spectrum of GeO_2[48].

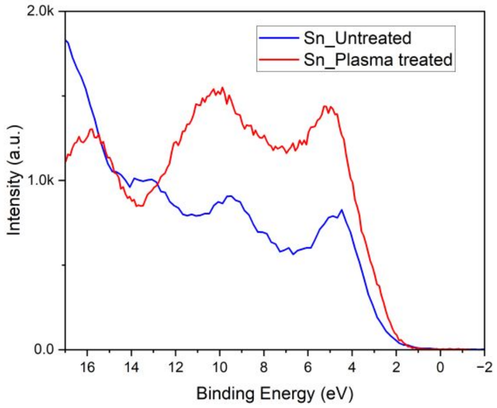

Figure S11. The valence band acquired from the as-deposited and plasma-treated PPX-$SnMe_3$. The spectrum patterns match with the trend in the SnO and SnO_2.

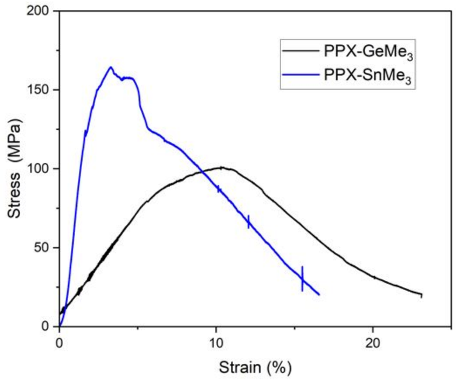

Figure S12. Tensile testing of new PPX films.

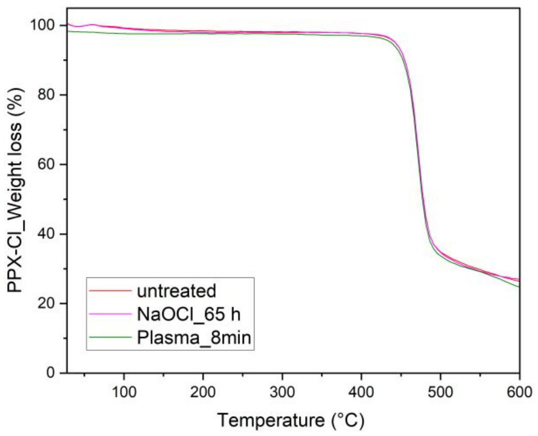

Figure S13. TGA spectra of untreated, NaOCl-, Plasma-treated PPX-Cl. The weight loss trends are identical across all treatments.

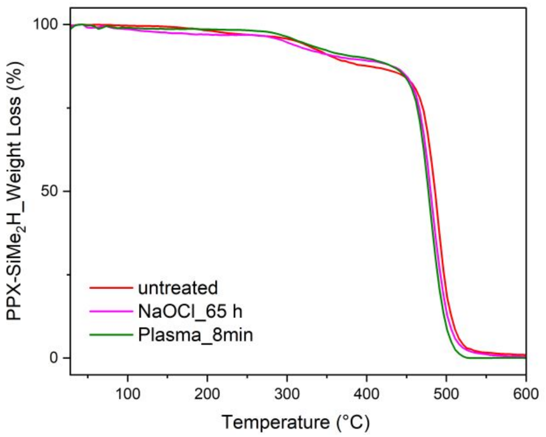

Figure S14. TGA spectra of untreated, NaOCl-, Plasma-treated PPX-$SiMe_2H$. The weight loss trends are identical across all treatments.

Table S1. Elemental analysis results of novel parylenes and the calculated theoretical oxygen rate.

	Element	Calculated Wt%	Obsereved Wt%
$GeMe_3$-cy (**1**)	C	59.82	60.69
	H	7.30	6.88
	Ge	32.88	24.72
	Total	100	92.29
$SnMe_3$-cy (**2**)	C	49.90	49.54
	H	6.04	6.06
	Sn	44.47	44.0
	Total	100	99.60
PPX-$GeMe_3$	C	59.82	53.81
	H	7.30	6.35
	Ge	32.88	27.30
	Total	100	87.46
PPX-$SnMe_3$	C	49.90	46.90
	H	6.04	5.15
	Sn	44.47	43.5
	Total	100	95.55

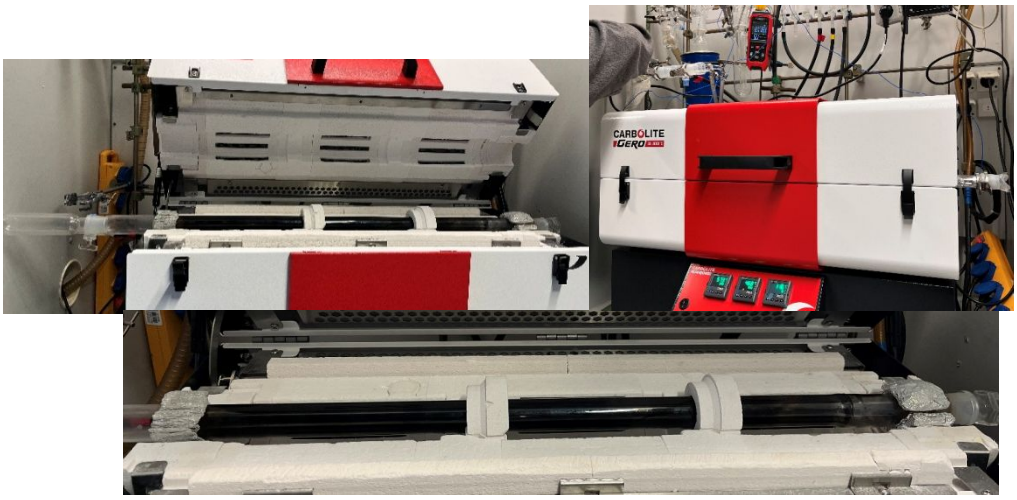

Figure S15. Experimental set up of the CVD polymerization: the tubular chamber is separated into three sections by ceramic block.

4,16-Bis(trimethylgermanyl)[2.2]paracyclophane	
CCDC number	2491478
Empirical formula	$C_{22}H_{32}Ge_2$
Formula weight	441.65
Temperature [K]	100(2)
Crystal system	triclinic
Space group (number)	$P\overline{1}$ (2)
a [Å]	9.7627(4)
b [Å]	10.1885(5)
c [Å]	11.4276(6)
α [°]	106.112(2)
β [°]	100.987(2)
γ [°]	100.545(2)
Volume [Å³]	1037.93(9)
Z	2
ρ_{calc} *[gcm⁻³]*	1.413
μ [mm⁻¹]	2.897
F(000)	456
Crystal size [mm³]	0.243×0.268×0.656
Crystal colour	colorless
Crystal shape	block
Radiation	MoK_α (λ=0.71073 Å)
2θ range [°]	3.84 to 54.33 (0.78 Å)
Index ranges	−12 ≤ h ≤ 12<br>−13 ≤ k ≤ 13<br>−14 ≤ l ≤ 14
Reflections collected	48231
Independent reflections	4592<br>R_{int} = 0.0423<br>R_{sigma} = 0.0195
Completeness to θ = 25.242°	100.0 %
Data / Restraints / Parameters	4592 / 0 / 223
Absorption correction T_{min}/T_{max} *(method)*	0.4617 / 0.7455 (multi-scan)
Goodness-of-fit on F^2	1.053
Final R indexes [I≥2σ(I)]	R_1 = 0.0185<br>wR_2 = 0.0453
Final R indexes [all data]	R_1 = 0.0206<br>wR_2 = 0.0465
Largest peak/hole [eÅ⁻³]	0.32/−0.57
